# Effects of repetitive twice-weekly transcranial direct current stimulations on fatigue and fatigability in people with multiple sclerosis

**DOI:** 10.1038/s41598-023-32779-y

**Published:** 2023-04-11

**Authors:** Stefanie Linnhoff, Aiden Haghikia, Tino Zaehle

**Affiliations:** 1 Department of Neurology, Otto-von-Guericke-University Magdeburg, Leipziger Street 44, 39120 Magdeburg, Germany; 2 Center for Behavioral Brain Sciences (CBBS), 39106 Magdeburg, Germany; 3 German Center for Neurodegenerative Diseases (DZNE), 39120 Magdeburg, Germany

**Keywords:** Diseases of the nervous system, Multiple sclerosis

## Abstract

Fatigue is associated with a dramatically decreased quality of life in people with multiple sclerosis (pwMS). It refers to a constant subjective feeling of exhaustion and performance decline, known as fatigability. However, inconsistency and heterogeneity in defining and assessing fatigue have led to limited advances in understanding and treating MS-associated fatigue. Transcranial direct current stimulation (tDCS) has emerged as a promising, non-pharmaceutical treatment strategy for subjective fatigue. However, whether repetitive tDCS also have long-term effects on time-on-task performance has not yet been investigated. This pseudorandomized, single-blinded, and sham-controlled study investigated tDCS effects on behavioral and electrophysiological parameters. 18 pwMS received eight twice-weekly 30 min stimulations over the left dorsolateral prefrontal cortex. Fatigability was operationalized as time-on-task-related changes in reaction time variability and P300 amplitude. Additionally, subjective trait and state fatigue ratings were assessed. The results revealed an overall decrease in subjective trait fatigue ratings that lasted at least four weeks after the stimulations. However, the ratings declined after both anodal and sham tDCS. No effects were found on subjective state fatigue and objective fatigability parameters. Linear Mixed Models and Bayesian Regression models likewise favored the absence of a tDCS effect on fatigability parameters. The results confirm the complex relationship between MS-associated fatigue and fatigability. Reliable and clinically relevant parameters need to be established to extend the potential of tDCS for treating fatigability. Furthermore, our results indicate that consecutive stimulations rather than twice-weekly stimulations should be the preferred stimulation scheme in future studies.

## Introduction

Fatigue is a challenging symptom of several neurological disorders. In contrast to tiredness, fatigue describes the overwhelming feeling of exhaustion, which manifests both cognitively and physically and does not resolve with rest or sleep. With up to 80% probability of occurrence, it is also one of the most common symptoms of multiple sclerosis^1^. MS is a primarily neuroinflammatory disease of the central nervous system with neurodegenerative features. Thus, symptoms vary according to the inflammatory lesion site. Fatigue, together with motor impairments, is considered the symptom that most reduces the quality of life of people with multiple sclerosis (pwMS)^2^ and constitutes the leading cause of early retirement^3^.

Developing efficient therapeutic methods for overcoming fatigue is of high clinical relevance. However, there is no general agreement on which treatment method is most effective for treating MS-associated fatigue. A non-invasive, non-pharmaceutical method that has been of interest in recent years is transcranial direct current stimulation (tDCS). During tDCS, a constant low-intensity electrical current is applied via two or more surface electrodes on the scalp resulting in modulation of cortical excitability. This is generally described via a shift in resting membrane potential that leads to depolarization (anodal) or hyperpolarization (cathodal) of neuronal membranes^4–6^. While the described modulation of cortical excitability is reversible^7^, other studies report long-term effects of tDCS via long-term potentiation^6,8^. Furthermore, these tDCS effects seem to depend on several parameters such as electrode-to-cortex distance, cerebrospinal fluid thickness, as well as the orientation of pyramidal neurons^9^. Due to fatigue-related functional abnormalities in the cortico-striato-thalamo-cortical network^10,11^, several studies investigated the efficacy of tDCS in restoring altered neuronal excitability in pwMS. The majority of those studies stimulated the left dorsolateral prefrontal cortex (DLPFC) for at least three or more consecutive sessions^12–18^ and reported positive effects on subjective fatigue ratings. Other studies improved subjective fatigue by stimulating the bilateral primary somatosensory cortex^19–21^ or the bilateral primary motor cortex^22,23^.

In recent years, however, there has been an increasing discussion that cognitive fatigue is characterized by an ongoing feeling of subjective exhaustion but also by an objectively measurable performance decline. While subjective fatigue can be subdivided into a trait (long-term) or state (momentary) component, objective fatigue is, per definition, state-dependent and refers to the failure to maintain one’s own individual optimal performance over time^24,25^. While subjective trait fatigue refers to an ongoing feeling which does not resolve with rest or sleep, the subjective feeling of momentary (state) exhaustion as well as the individual’s cognitive performance fluctuates significantly during the day, typically peeking in the late afternoon^26,27^. Additionally, Dettmers et al. recently reported that cognitive fatigability rather than subjective fatigue predicts the employment status of pwMS^28^. According to a model proposed by Hanken et al., subjective fatigue results from activated immune-to-brain pathways, innervating interoceptive and homeostatic brain areas, leading to the subjective feeling of fatigue^29^. Furthermore, this results in increased interoceptive interference and distracts cognitive processes, manifesting in performance decline. However, performance decrements in alertness or vigilance tasks may also result from MS-induced focal brain atrophy, especially in frontal areas^29,30^. It is still an open question whether subjective fatigue and fatigability in pwMS are associated or independent from one another.

In healthy participants, anodal tDCS over the left DLPFC prevented vigilance decrements in sleep-deprived participants and improved subjective fatigue ratings^31–33^. Likewise, healthy participants maintained and even improved their working memory performance in an hour-long two-back task while receiving anodal tDCS compared to sham stimulation^34^. In pwMS, anodal stimulation led to decreased reaction times with time-on-task, but only in participants suffering from mild to moderate cognitive fatigue^35^. Fiene et al. repetitively assessed reaction times as well as P300 amplitudes and latencies while they applied either anodal or sham tDCS in pwMS^36^. The authors report that anodal, compared to sham stimulation, counteracted fatigability-related cognitive exhaustion resulting in greater P300 amplitudes and a reduced increase in P300 latency and reaction times in pwMS. However, both studies reported that stimulation did not counteract the increase in subjective state fatigue ratings, despite the sustained or improved behavioral performance. The relationship between subjective fatigue and fatigability is still a topic of controversy. Thus, in numerous studies the increase in subjectively perceived fatigue is not paralleled by an objectively measurable performance decline and vice versa^37^. This lack of association between subjective fatigue and fatigability complicates the evaluation of tDCS effects, particularly when improvement in subjective feelings is the primary clinical concern, whereas objectively measurable effects improve the evaluation of tDCS as an alternative treatment option. The previous literature has shown that a single session of tDCS can improve fatigability-related performance decrements in pwMS. In contrast, multiple repetitive tDCS sessions are necessary to induce subjectively perceivable changes in the global feeling of fatigue^37^.

Accordingly, the current study investigates the effects of repetitive twice-weekly anodal tDCS sessions on objective fatigability development as well as subjective state fatigue ratings in pwMS. To our knowledge, this is the first study to explore tDCS effects on fatigability after repetitive stimulations in pwMS. We hypothesized that repetitive tDCS would reduce subjective fatigue ratings and the fatigability-related performance decline with time-on-task.

## Methods

### Participants

We enrolled 18 participants (male = 3) aged 23 to 65 years in this study. All participants were diagnosed with clinically definite MS according to the McDonald criteria and were native German speakers. All participants had relapsing–remitting MS. Baseline group characteristics are listed in Table 1.

**Table 1:** Baseline group characteristics.

	Anodal group (*n* = 9) mean ± *SD*	Sham group (*n* = 9) mean ± *SD*	
Gender f/m	8/1	7/2	
Age [years]	40.44 ± 14.37	40.89 ± 10.49	*p* = 0.825
Disease duration [years]	5.22 ± 4.55	8.44 ± 8.81	*p* = 0.350
EDSS [points]	3.00 ± 1.79	2.78 ± 1.66	*p* = 0.718
BDI-FS [points]	2.33 ± 0.71	2.33 ± 2.69	*p* = 0.339
WEIMuS_tot_ [points]	33.89 ± 10.37	44.44 ± 9.79	*p* = 0.093
WEIMuS_cog_ [points]	18.44 ± 5.05	23.00 ± 4.47	*p* = 0.092
WEIMuS_phy_ [points]	15.44 ± 6.15	21.44 ± 6.29	*p* = 0.132
Cross-over participation	4	5	

*BDI-FS* Becks Depression Inventory Fast Screen, *EDSS* Expanded Disability Status Scale, *WEIMuS* Wuerzburg Fatigue Inventory for Multiple Sclerosis.

Inclusion criteria were a minimum of 3 months since the last relapse or use of corticosteroids, no paresis of the upper limb, no previous or current neurological or psychiatric comorbidities, and no treatment with fatigue medication. Participants neither had a diagnosed depression nor pharmacological treatment with antidepressants. The disease-modifying MS therapy consisted of glatiramer acetate (*n* = 5), interferon-beta (*n* = 3), fingolimod (*n* = 3), teriflunomide (*n* = 2), and dimethyl fumarate (*n* = 1). Four participants had no MS medication. Additionally, participants had to meet tDCS criteria such as no cardiac arrhythmias or pacemaker, no pregnancy, no metal in the cranium except in the mouth (retainer), no surgical clips in or near the brain, no epilepsy, or epileptic seizures in the lifetime, no recurring unexplained blackouts, and no chronic skin diseases on the shoulder, face, and scalp. All participants reported having normal hearing and normal or corrected-to-normal vision. They were recruited from the outpatient pool of the University Hospital of Magdeburg and received monetary reward (Euro 80 in total) for participation in the study. The study was approved by the local ethics committee of the University Clinic of Magdeburg and was conducted in accordance with the Declaration of Helsinki. All participants gave written informed consent before participation.

### Procedure

We designed a placebo-controlled study in two phases to assess the efficacy of anodal tDCS over the left DLPFC. In “Phase I”, we used a between-subject design. The first ten patients were randomly allocated using blocked randomization at a 1:1 ratio to an anodal or sham tDCS group using a web-based random number generator. Simple randomization was used, with no stratification. After that, eligible participants were pseudo-randomly assigned in a repeating sequence. After completing the first phase, nine participants (four from anodal, five from sham group) agreed to participate in “Phase II”, in which they crossed groups and participated a second time after a 12 weeks wash-out interval.

**Figure 1:**
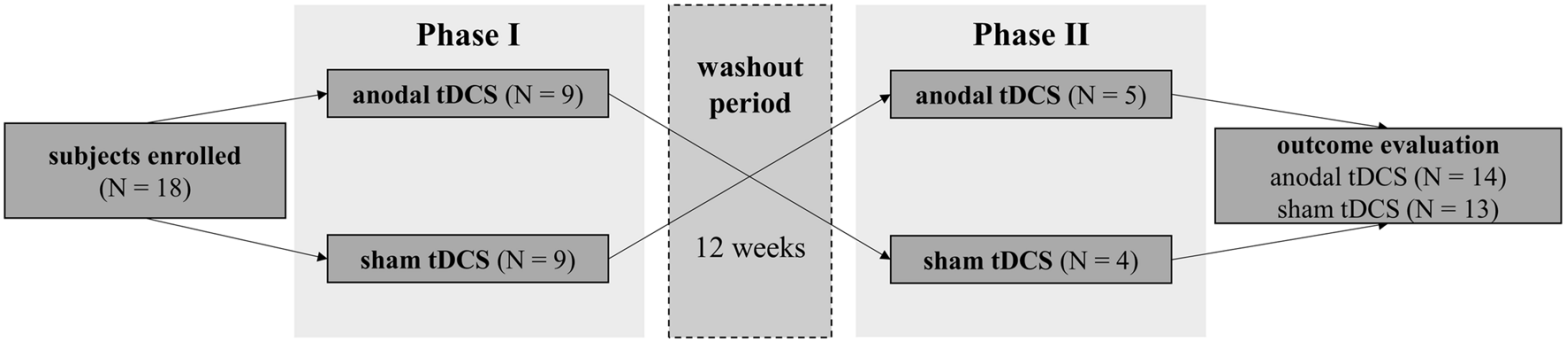
Schematic design of the study. A two-phased, randomized controlled, cross-over study with two groups [anodal and sham tDCS (transcranial direct current stimulation)].

Each phase consisted of three experimental sessions in which subjective fatigue and fatigability scores were assessed. In between pre- and post-session, eight anodal stimulation sessions were administered. A follow-up session took place four weeks after the post-session. The study design is illustrated in Fig. 1.

**Figure 2:**
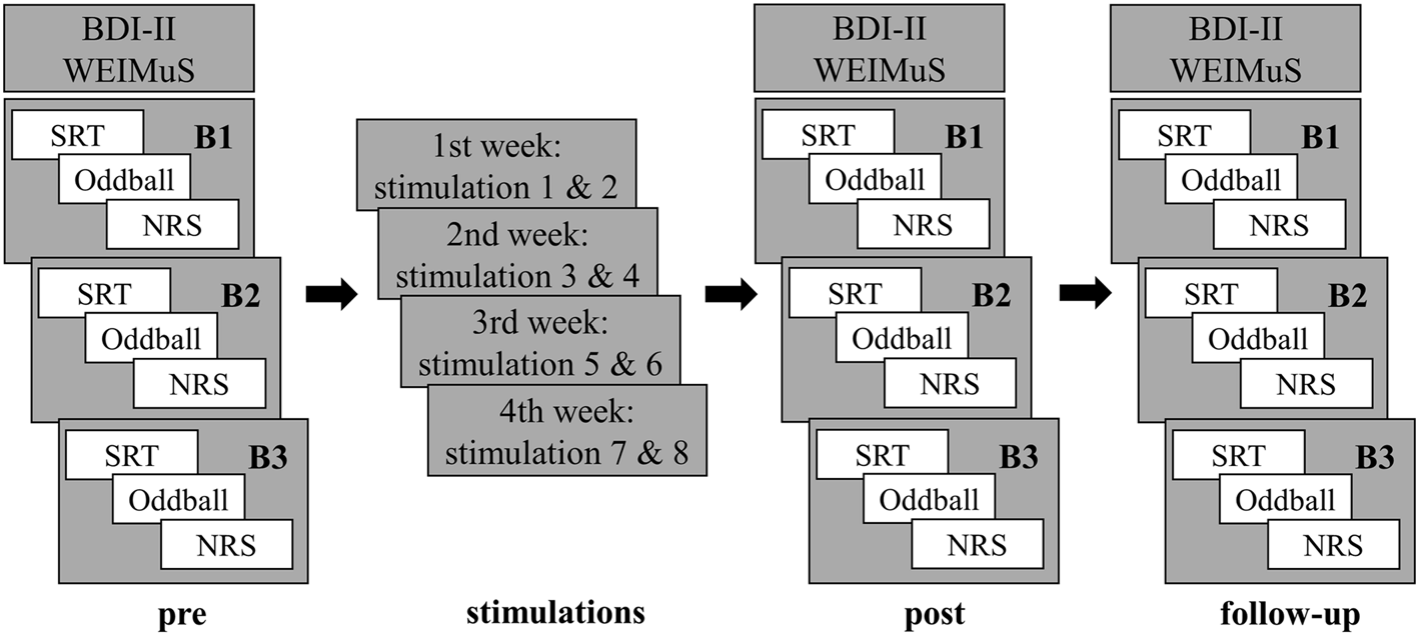
Experimental procedure. The experiment consisted of three experimental sessions with three test blocks each (B1, B2, and B3). In each test block, fatigability and subjective state fatigue scores were assessed. RT variability was evaluated via a serial reaction time task (SRT), P300 during an auditory oddball task, and subjective state fatigue via a 10-point numerical rating scale (NRS). Additionally, depression scores using the Becks Depression Inventory (BDI-II) as well as subjective trait fatigue scores using the Wuerzburger Fatigue Inventory (WEIMuS) were assessed. In between pre- and post-session, eight anodal or sham stimulation sessions were administered.

Initially, participants signed informed consent and completed several questionnaires to assess their disease history and their ability to participate in the experiment (tDCS questionnaire). At the beginning of each session, participants rated their current mood (BDI-II) and subjective trait fatigue (WEIMuS)^38^. The WEIMuS consists of 17 items on a five-point Likert scale from “almost never” to “almost always” and evaluates a total score, as well as individual scores for the physical and cognitive fatigue dimensions. Higher scores reflect a stronger fatigue expression. Subsequently, the electroencephalogram (EEG) was prepared and the session started. Each experimental session (pre, post, and follow-up session) consisted of three test blocks. Each test block (B1, B2, and B3) consisted of three tasks: a serial reaction time task (SRT), an auditory oddball task (see below), and a 10-point numerical rating scale (NRS), where participants rated their current feelings of fatigue depending on “how mentally exhausted” they felt at the time from 0 (not at all) to 10 (extremely exhausted). Test blocks were repeated three times in order to assess time-on-task changes relative to the respective individual and day-dependent baseline value (performance at B1). All three experimental sessions took place at the same time of day. The subjects were seated in an upright position in front of a computer screen (60 cm distance). Between the tasks, the subjects could take breaks. The experimental design of each phase is illustrated in Fig. 2.

### Experimental tasks

Behavioral performance was evaluated using the SRT task adapted from Woods et al.^39^. Thus, participants were asked to press the space bar with their dominant hand as quickly as possible when a target in the form of a bulls-eye stimulus was presented on the computer screen. One test block consisted of 120 stimuli and lasted approximately 4.5 min. The interstimulus intervals ranged from 1000 to 2000 ms in 250 ms steps, and they were pseudorandomly used with equal probability. The stimulus presentation lasted 200 ms. The bulls-eye had a diameter of 5.72° of visual angle and was presented in black color on a white screen. Between stimuli, a fixation cross was shown.

To evoke the P300, we utilized an acoustic oddball paradigm. Therefore, a randomized series of frequent standard tones (1000 Hz, 80%) and deviant “target” tones (2000 Hz, 20%) were presented. Both stimuli had a presentation time of 70 ms (10 ms rise/fall time) and were presented binaurally via headphones [Sennheiser HD 65 TV] at 70 dB. The interstimulus interval varied between 1500 and 2000 ms. During the task, participants were instructed to press the space bar when the target tone was presented and to ignore other stimuli. One test block consisted of 240 standard and 60 deviant tones and lasted approximately 10 min with a 1-minute break. Both tasks were presented using the Presentation software [Neurobehavioral Systems Inc, USA].

### tDCS design

The anodal tDCS group received eight sessions of active treatment, while the sham tDCS group received placebo treatment. The stimulations were administered two times a week for four weeks, and at least one day of rest was required between sessions. The stimulation sessions took place in the clinic. During the stimulation sessions, participants were seated in an upright position and at rest. A battery-driven DC stimulator [DC Stimulator Plus, NeuroConn, Germany] delivered the stimulation using two rubber electrodes covered with saline-soaked sponges. The anode (5 × 7 cm) was placed over the left DLPFC (F3 according to the international 10–20 system for EEG electrode placement). The reference electrode (5 × 10 cm) was placed extracephalically over the right shoulder to prevent unwanted cephalic polarization effects under the return electrode^40^. For the anodal tDCS condition, a direct current with an intensity of 1.5 mA was applied for 30 min stimulation with a 15 s fade in/out time. For the sham tDCS condition, we used the 15 s fade in – 30 s stimulation – 15 s fade out approach suggested by Ambrus et al. to simulate skin sensations and ensure blinding^41^. The impedance of stimulation electrodes was kept below 10 kΩ.

At the end of every stimulation session, participants were asked to fill out a short questionnaire on tDCS side effects. The questionnaire was designed according to the consensus tDCS guidelines^42^ and asked whether and to what extent participants felt any of the following sensations due to stimulation: headache, nausea, dizziness, loss of concentration, fatigue, metallic taste, skin irritation or itch, tickle or heat on the scalp. The numeric rating scale ranged from 0 = no sensation to 3 = strong sensation.

### EEG signal recording and preprocessing

EEG was recorded at Cz, Pz, POz, P3, PO3, P4, and PO4 using Ag/AgCl-electrodes mounted in an elastic cap [EasyCap GmbH, Germany]. The ground electrode was attached to the AFz position, and all channels were referenced to the nasion. The electrooculogram (EOG) was recorded using two electrodes placed below the pupil (vertical EOG) and to the external canthus of the left eye (horizontal EOG). The data was recorded by Brain DC amplifier [Brain Products, Germany] and the corresponding software [BrainVision Recorder, Version 1.20, Brain Products, Germany] sampled at 1000 Hz. Impedances were kept below 5 kΩ. EEG preprocessing and data analysis were carried out in BrainVision Analyzer [Version 2.2.2, Brain Products GmbH, Gilching, Germany]. The EEG data were off-line band-pass filtered from 0.1 to 30 Hz and corrected for eye-movement artifacts using the Gratton and Coles method^43^. Using automatic artifact rejection, epochs with amplitudes exceeding ± 75 μV, voltage steps greater than 100 μV, or an absolute difference of 200 μV between the minimum and maximum voltage within 200 ms intervals were excluded. For P300 analysis, trials in which participants gave the correct answer were segmented into epochs from − 200 ms to 800 ms relative to stimulus onset. The 200 ms pre-stimulus interval served as a baseline. Averages for the standard and deviant tones were computed separately for each block, session, and condition. The final peak detection was performed on single-subject deviant waves, and the P300 was quantified as the mean amplitude between 250 and 450 ms at electrode Pz.

### Statistical analysis

To explore blinding success, we analyzed the tDCS questionnaires by summing up all ratings of the eight stimulation sessions and checking for group differences using the Mann–Whitney *U* test (Phase 1) and differences between the phases using the Wilcoxon-signed-ranked test (Phase II).

Data of all participants were analyzed using linear mixed models (LMMs). This way, we accounted for the unbalanced study design as well as the non-independence of the data. We used R Statistical Software (version 4.2.0, R Core Team, 2022) for statistical analyses and production of all plots. LMMs were performed using the lmer function from the *afex* package^44^. P values were obtained using Sattersthwaite’s approximation method. For subjective trait fatigue scores, we used the items of the cognitive dimension of the WEIMuS questionnaire (WEIMuS_cog_). To assess objective fatigability, we used reaction time variability (RT variability) and P300 peak amplitudes. Subjective state fatigue was evaluated via the NRS ratings. We computed delta scores for the three time-on-task parameters by subtracting scores of block B3 from baseline responses in block B1. This improved the model fit and met the fatigability definition from Kluger et al. according to which fatigability is defined as a performance decline with time-on-task^25^. RT variability was analyzed using the standard deviation of reaction times. Subjective data, RT variability, and P300 amplitudes were considered as dependent variables. *Session* (pre, post, follow-up) and *group* (anodal, sham), as well as their interaction, were considered as fixed factors. Data from the sham group at pre session were used as baseline. Subjects were used as random effects, thus accounting for the individual specific characteristics and the dual participation of some subjects resulting from the cross-over design. Furthermore, to account for order effects, we also included *order* as a covariate and compared both models using the Akaike Information Criterion (AIC). However, *order* as an additional factor did not improve any of the models. Thus, it was not included in the final model. We further used Bayesian Linear Regression Models using the *brms* package^45^ with the same model formula and default priors to further evaluate our results when LMMs yielded no effect of tDCS on the fatigability parameters.

## Results

### tDCS adverse effects and blinding

All participants tolerated the stimulation well. No participant reported severe side effects or pain under the electrodes. In Phase 1, there were significant differences between anodal and sham group concerning tingling (*p* = 0.006), skin redness (*p* = 0.004), and itching under the electrode (*p* = 0.040). In all cases, this resulted from stronger feelings of the respective symptom in the anodal compared to the sham group. However, no participant reported having been aware of the stimulus condition when being asked at the end of the study. In Phase II, there are significant differences between the conditions concerning tingling (*p* = 0.042) and skin redness under the electrode (*p* = 0.036). Again, this resulted from higher ratings after anodal compared to sham stimulations. Finally, in Phase II, we asked participants when they thought they had received anodal and when sham stimulation. Two (2/9) participants guessed the order right, two (2/9) were not sure, and five (5/9) thought they received stimulations in the opposite order.

### Subjective fatigue

#### Subjective trait fatigue

**Figure 3:**
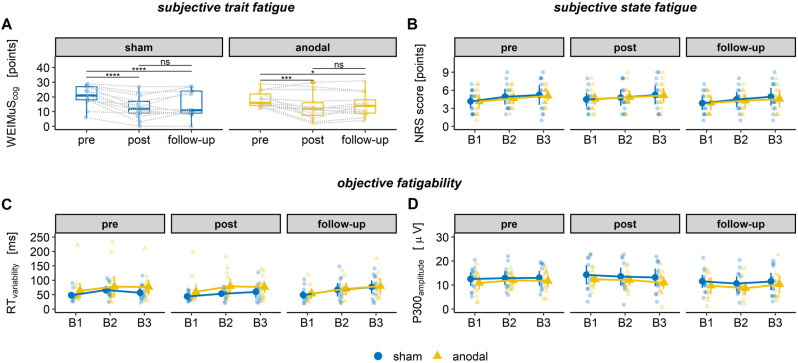
tDCS effects on fatigue and fatigability parameters. (**A**) WEIMuS cognitive scores as a function of session (pre, post, follow-up) separate for sham and anodal group. (**B**–**D**) Changes in numerical rating scores (NRS, **B**), reaction time variability (RT variability, **C**), and P300 amplitudes (**D**) as a function of block (B1, B2 and B3) (mean ± CI).

Changes in subjective trait fatigue ratings (WEIMuS_cog_) are shown in Fig. 3A. The model to predict the subjective cognitive fatigue ratings showed a significant effect of *session* [*F*(2, 57.12) = 26.824, *p* < 0.001, ${\eta }_{p}^{2}$ = 0.48] but no significant effect of *group* [*F*(1, 61.20) = 0.089, *p* = 0.767] and no significant interaction of *session* and *group* [*F*(2, 57.12) = 1.186, *p* = 0.313]. Thus, cognitive fatigue scores generally decreased after repetitive stimulations. The initial ratings of trait fatigue were 20.64 points [β_intercept_, 95% CI (16.96, 24.30)] in the sham group and 19.30 points (β_intercept_ + β_group_) in the anodal group. After stimulations, fatigue was reduced by 7.69 points in the sham group [β_session_, 95% CI (− 10.20, − 5.19)] and by 5.07 points in the anodal group (β_session_ + β_session*group_). Bonferroni-corrected post hoc comparisons revealed significant reductions in fatigue scores from pre to post session (*p* < 0.001) and from pre to follow-up session (*p* < 0.001). There were no further reductions from post to follow-up session (*p* = 0.151) indicating that reduced fatigue scores remained stable.

#### Subjective state fatigue

The subjective state fatigue ratings as a function of time-on-task separate for both groups and the three sessions are shown in Fig. 3B. They increased by about one point during the presession in both groups (sham: 1.08 ± 1.26; anodal: 0.93 ± 1.07). In contrast, during the post session, the ratings increased by about 0.77 points (± 1.17) in the sham group and by only 0.57 points (± 1.28) in the anodal group. Similar results were found during the follow-up session (sham: 1.08 ± 1.26; anodal: 0.67 ± 1.36). However, the LMM to predict subjective state fatigue changes showed no significant effects of *session* [*F*(2, 55.92) = 0.805, *p* = 0.452] nor of *group* [*F*(1, 68.98) = 0.275, *p* = 0.602] and no significant interaction [*F*(2, 55.92) = 0.089, *p* = 0.915]. Consequently, the repetitive anodal stimulation appeared not to have affected the subjective fatigue increase during the performance of an exhaustive task. To further explore this, we additionally subjected the data to a Bayesian regression model using the same model formula. The Bayesian model revealed that the interaction of *session* (pre, post) and *group* (sham, anodal) has a probability of only 52.28% of being negative [Median = -0.03, 95% CI (-1.13, 1.05)], which would indicate a reduced increase of subjective state fatigue in the anodal group during the post session compared to the sham group and pre session. Additionally, this interaction's significance is considered highly uncertain (18.61% in ROPE). Thus, this further supports the evidence favoring an absent tDCS effect on subjective state fatigue with time-on-task.

### Objective fatigability parameters

#### Reaction time variability

RT variability similarly increased in both groups during all three sessions. Mean delta scores during pre session are 9.28 ms (± 13.16 ms) in the sham group and 12.98 ms (± 15.97 ms) in the anodal group. During the post session, RT variability increased by 15.92 ms (± 21.04 ms) in the sham group and by 16.54 ms (± 34.29 ms) in the anodal group. In contrast, during the follow-up, RT variability increased by 27.79 ms (± 31.50 ms) in the sham group and 26.18 ms (± 43.48 ms) in the anodal group. Consequently, the LMM to predict changes in RT variability with session and group showed no significant results [session: *F*(2, 55.92) = 2.645, *p* = 0.080; group: *F*(1, 71.05) = 0.130, *p* = 0.720; session x group: *F*(2, 55.92) = 0.076, *p* = 0.927]. We again performed a Bayesian regression model to evaluate the results further. The interaction of *session* (pre, post) and *group* (sham, anodal) has a probability of only 60.42% of being negative [Median = -3.24, 95% CI (-30.24, 23.61)], which would indicate that the RT variability increase during the post session is reduced in the anodal group compared to the sham group and the pre session. This interaction’s significance is considered highly uncertain (16.53% in ROPE). Consequently, an absent tDCS effect on RT variability during an exhaustive task is more likely. Figure 3C depicts the RT variability as a function of time-on-task separate for both groups and the three sessions.

#### P300 peak amplitude

P300 peak amplitudes increased during the pre session in the sham group (0.53 μV ± 2.86 μV) and the anodal group (0.81 μV ± 3.65 μV). In contrast, during the post session, they decreased in both groups (sham: -1.09 μV ± 4.60 μV; anodal: − 1.37 μV ± 3.11 μV). During the follow-up, peak amplitudes decreased in the sham group (− 0.05 μV ± 3.76 μV), while they increased in the anodal group (0.55 μV ± 5.01 μV). The LMM to predict the change in P300 peak amplitude with session and group showed no significant effects [session: *F*(2, 55.62) = 2.601, *p* = 0.083; group: *F*(1, 69.98) = 0.078, *p* = 0.781] and no significant interaction [*F*(2, 55.62) = 0.154, *p* = 0.857]. The Bayesian regression model revealed that the interaction of *session* (pre, post) and *group* (sham, anodal) has a probability of only 63.70% of being negative [Median = -0.63, 95% CI (-4.17, 2.89)] and the significance of this interaction is considered as highly uncertain (16.89% in ROPE). Consequently, as in the other fatigability parameters, this supports the conclusion that twice-weekly repetitive tDCS sessions did not affect P300 peak amplitude changes with time-on-task. P300 peak amplitude values as a function of time-on-task separate for both groups and the three sessions are shown in Fig. 3D.

## Discussion

This study investigated the effects of multiple, twice-weekly tDCS sessions on both subjective fatigue and objective fatigability parameters in people with MS-associated fatigue. Subjective trait fatigue ratings decreased significantly after both anodal and sham tDCS. However, we did not observe tDCS-specific effects on subjective state fatigue ratings or objective fatigability parameters.

### Placebo effect

Our observed improvement of subjective trait fatigue after anodal stimulations is in line with a series of previous studies^12–16,18–23^. However, our study showed an improvement in subjective trait fatigue independent of the stimulation condition. This effect of sham stimulation might be related to a marked placebo effect. While this has not been systematically investigated in most previous tDCS studies, at least one study reported similar placebo effects^17^. Saiote et al. stimulated the DLPFC for 20 min on five consecutive days. Similar to our results, both the sham and the anodal condition significantly reduced fatigue ratings. According to Saiote et al. methodological problems with self-report instruments may have contributed to the pronounced placebo effects during the sham condition and, in consequence, to a partial masking of the anodal tDCS effect. However, we used the WEIMuS questionnaire, which has been validated in a large MS sample and discriminates successfully between MS patients with and without fatigue. Additionally, the questionnaire considers a time window of 2 weeks and was, therefore, suitable for our experimental design.

However, it is noteworthy that the repetitive stimulation design required intense caregiving over a significant amount of time, including conversations and symptom exchange. This likely resulted in a stimulation-independent, positive interaction between the experimenter and the participants that, in turn, had a significant impact on the participants’ subjective perception, further promoting the observed placebo effect.

### Objective fatigability parameters

We decided to use RT variability and P300 amplitude as objective fatigability parameters because they are easy and reliably assessed. Especially the P300 event-related potential is widely used as an index of cognitive functioning. In pwMS, previous studies already demonstrated longer P300 latencies and smaller amplitudes associated with subjective trait fatigue^36,46,47^. Additionally, tDCS counteracted those fatigue-induced changes in pwMS^36^ and other clinical cohorts^48,49^. On the other hand, higher subjective state fatigue ratings in healthy participants did not affect cognitive performance and had no impact on P300 components^50^. Up to now, there is no consensus concerning clinically relevant and reliable outcome measurements for fatigability in MS, despite a wide variety of studies addressing this research question. Other electrophysiological parameters such as sensory and sensorimotor gating or spectral power changes in the alpha and theta band may be more suitable^51–53^.

Furthermore, we investigated all parameters over a prolonged period of three blocks and assessed changes compared to baseline values. We assumed that subjective fatigue ratings and RT variability would increase with time-on-task, while P300 amplitudes would decrease, as shown previously^26,36^. However, in the present study, we did not observe robust results but instead revealed highly heterogeneous data patterns that differed inter- and intraindividually. Thus, while all participants reported an increase in subjective exhaustion, indicating that the chosen tasks effectively induced cognitive fatigability, they were mostly able to uphold their cognitive performance. A better approach to study fatigability development in the future might be to measure the changes in performance during sustained mental effort. Hence, to compare performance at the beginning and the end of an ongoing cognitively demanding task. Behavioral changes associated with an increase in subjective exhaustion have repeatedly been observed in studies using this approach^35,54,55^. Nevertheless, the literature remains heterogeneous regarding the existence of valid and reliable objective fatigability parameters^37^.

### Stimulation design

We selected the stimulation parameters based on the results of previous studies on fatigue in pwMS. As in our study, the majority of these investigations stimulated with a current intensity of 1.5 mA^17,19,20,22,35,36^. However, it should be noted that there are also studies that showed successful results with 2 mA stimulation intensities^13,15,16,18^. Notably, Charvet et al. reported a greater reduction of fatigue ratings after 2 mA compared to 1.5 mA^18^. Furthermore, we chose 30 min as stimulus duration based on previous results from Fiene et al. who showed that a single session of 30 min tDCS stimulation had positive effects on fatigability in people with MS^36^. However, there is an ongoing discussion whether 20 min might be the more optimal stimulus duration^56^. To date, the optimal stimulation parameters to improve fatigue and fatigability in pwMS are still under debate and need to be further investigated in future studies (i.e. polarity, electrode locations and mounting, session number and duration, current intensity).

Furthermore, the stimulation design of the present study differed from previous investigations. We administered stimulations offline and twice-weekly for four weeks, rather than in a single online session^35,36^ or on consecutive sessions^12,14,16–21,23^. Accordingly, our observations could be partly attributed to our non-daily stimulation design. Alonzo et al. examined the influence of daily vs. second daily stimulations on motor evoked potentials^57^. They reported that daily tDCS sessions lead to a more significant increase in neuronal excitability than second daily tDCS. However, as a result of their physical impairment and chronic fatigue, pwMS often refrain from participating in time-consuming study protocols. Hence, when planning the study design, attention was paid to the study’s external validity and practicability. In addition, Mortezanejad et al. treated pwMS with six second-daily stimulations^58^. They found a significant reduction in fatigue ratings and an increase in quality of life, while there were no effects after sham stimulation. Likewise, To et al. used a stimulation design similar to ours^59^. They applied eight anodal stimulations over the left DLPFC for 20 min and reported positive effects on subjective fatigue ratings in people with fibromyalgia.

Additionally, we assessed all performance parameters before and after the offline stimulations, in which the participants were at rest, sitting in a comfortable chair in a quiet room. In contrast, other studies evaluated tDCS-related changes during or immediately following a single stimulation^35,36^. Especially concerning the effects of tDCS on fatigability, this might have led to a crucial disadvantage in this study. Thus, Dedoncker et al. systematically reviewed tDCS studies over the DLPFC and reported that online task performance while the stimulation is applied results in greater performance improvements compared to offline task performance^60^. However, the studies included in this meta-analysis only applied stimulations during a single session. The lack of offline advantages might have been compensated by repetitive stimulation and its cumulative effects^13,15,18^. Additionally, for a clinical application, it is imperative to achieve long-term effects. Single tDCS sessions while performing a cognitively demanding task lose external validity and may only provide temporary improvements. Stimulations studies combined with cognitive training during the stimulations sessions might lead to cumulative effects and long-term fatigability improvements in pwMS^18,61^. However, this has yet to be systematically evaluated in future studies focusing on fatigability improvement in pwMS.

### Limitations and perspectives

This study has several limitations. First, an interpretation of the results is limited by the small sample size and heterogeneous MS cohort – despite the careful inclusion of pwMS with similar disability status. Even though the majority of former studies on that topic used similar sample sizes, the results of our study cannot be generalized beyond the sample and must instead be considered as preliminary data. Furthermore, we decided to limit exclusion criteria to a minimum. While this improved external validity, it may have prohibited the emergence of any positive tDCS effects on fatigability. Thus, we included pwMS with elevated BDI values as long as they did not have a diagnosed depression or took antidepressants. Notably, there were only two participants with higher scores than the cutoff values, and these individuals were designated, by chance, to each of both groups. Therefore, it is unlikely that this influenced our results. Moreover, we did not assess sleep quality, which limits the results of our study. Since MS fatigue has been linked to quality of sleep^62^ it would have been desirable to control for effects on sleep quality.

Second, the study is limited by the lack of functional or structural neuroimaging data. In recent years, an increasing number of studies have emerged showing interindividual variability of tDCS effects, limiting the efficacy of brain stimulation^63–65^. Mosayebi-Samani et al. investigated the association between individual anatomical parameters and tDCS-induced electric fields and explored which parameters predicted the physiological outcome^63^. The authors found an association between electrical field values and cerebrospinal fluid thickness and electrode-to-cortex distance; in addition, the parameters predicted physical outcomes. Furthermore, lesion load predicts tDCS effects on fatigue in pwMS^17^. Ideally, future studies should pay more attention to these interindividual anatomical and clinical factors and focus more on personalized electrodes^19–21^.

Finally, we included participants with at least a minimum degree of subjective trait fatigue. A more appropriate approach might have been to include participants according to their degree of fatigability. Thus, assuming that in pwMS, subjective fatigue and fatigability can jointly appear but also exist independent of one another^29,37^, we cannot be sure that our sample initially suffered from fatigability. Future studies should pay more attention to this and explicitly investigate tDCS effects on participants with predetermined fatigability. Unfortunately, our sample size was too small to distinguish between high and low fatigability participants. However, reliable and valid objective parameters are required for this to be successful. And those have yet to be established.

## Conclusions

In this study, we investigated the effects of repetitive twice-weekly tDCS sessions on fatigue and fatigability in pwMS. Our results show a positive effect on subjective trait fatigue scores that lasted at least four weeks after the stimulations. However, this effect was independent of the stimulation scheme. No effects were observed on fatigability with time-on-task or subjective state fatigue scores. To date, there is no consensus about the relationship between subjective trait fatigue and objectively measurable fatigability. Our study once again demonstrates the complex relationship between MS-associated fatigue and fatigability. Improving subjective fatigue should continue to be the focus in daily clinical practice. However, especially in the context of the increasing number of people with fatigue, as currently observed by *Long Covid*^66^, it is imperative to extend the current subjective fatigue diagnosis with objective parameters for a more holistic approach and to broaden its acceptance. Considering that there is no effective treatment of MS-related fatigue available and that tDCS is easy to apply and well-tolerated, even the demonstration of a low degree of fatigue relief in a minority of patients will substantially improve healthcare in pwMS suffering from fatigue. However, future studies should prefer repetitive stimulation sessions on consecutive days instead of a twice-weekly stimulation scheme.

## Data Availability

The datasets generated and/or analyzed during the current study are available from the corresponding author on reasonable request.
